# The effect of patellofemoral pain syndrome on patellofemoral joint kinematics under upright weight-bearing conditions

**DOI:** 10.1371/journal.pone.0239907

**Published:** 2020-09-30

**Authors:** Jae-suk Yang, Michael Fredericson, Jang-Hwan Choi

**Affiliations:** 1 Division of Mechanical and Biomedical Engineering, College of Engineering, Ewha Womans University, Seoul, Korea; 2 Division of Physical Medicine and Rehabilitation, Department of Orthopedic Surgery, Stanford University, Stanford, California, United States of America; Texas State University, UNITED STATES

## Abstract

Patellofemoral pain (PFP) is commonly caused by abnormal pressure on the knee due to excessive load while standing, squatting, or going up or down stairs. To better understand the pathophysiology of PFP, we conducted a noninvasive patellar tracking study using a C-arm computed tomography (CT) scanner to assess the non-weight-bearing condition at 0° knee flexion (NWB0°) in supine, weight-bearing at 0° (WB0°) when upright, and at 30° (WB30°) in a squat. Three-dimensional (3D) CT images were obtained from patients with PFP (12 women, 6 men; mean age, 31 ± 9 years; mean weight, 68 ± 9 kg) and control subjects (8 women, 10 men; mean age, 39 ± 15 years; mean weight, 71 ± 13 kg). Six 3D-landmarks on the patella and femur were used to establish a joint coordinate system (JCS) and kinematic degrees of freedom (DoF) values on the JCS were obtained: patellar tilt (PT, °), patellar flexion (PF, °), patellar rotation (PR, °), patellar lateral-medial shift (PT_x_, mm), patellar proximal-distal shift (PT_y_, mm), and patellar anterior-posterior shift (PT_z_, mm). Tests for statistical significance (p < 0.05) showed that the PF during WB30°, the PT_y_ during NWB0°, and the PT_z_ during NWB0°, WB0°, and WB30° showed clear differences between the patients with PFP and healthy controls. In particular, the PF during WB30° (17.62°, extension) and the PT_z_ during WB0° (72.5‬0 mm, posterior) had the largest rotational and translational differences (JCS Δ = patients with PFP—controls), respectively. The JCS coordinates with statistically significant difference can serve as key biomarkers of patellar motion when evaluating a patient suspected of having PFP. The proposed method could reveal diagnostic biomarkers for accurately identifying PFP patients and be an effective addition to clinical diagnosis before surgery and to help plan rehabilitation strategies.

## Introduction

Approximately 25% of patients presenting with knee pain to musculoskeletal clinics are diagnosed with patellofemoral pain (PFP) [1]. Although extensively studied, the precise cause of PFP has not yet been entirely resolved because of the complexity of the interactions of biomechanical factors that can influence the patellofemoral joint (soft tissue and bone) [2]. Clinically, PFP is a symptom of excessive loading and often, abnormal motion of the patella (patellar maltracking) [3].

Typically, half of PFP patients are imprecisely diagnosed with patellar maltracking [4] based on the lateral translation of the patella in full knee extension. To prevent this kind of wrong diagnosis, we need to know the differences in the knee motion between patients and healthy people. Especially, we can distinguish patellar maltracking by lateral translation of the patella and other various features because the cause of PFP differs in patients with various features. Hence, additional tools are needed to easily distinguish patellar maltracking by lateral translation of the patella from the other features [5, 6].

Patellar tracking is typically performed by measuring the physical motion of the patella in the upright, squatting, and supine positions [7, 8]. Patellar tracking provides useful information during weight-bearing (WB) activities and can allow accurate diagnosis of PFP to ensure appropriate treatment. If the direct patellar tracking test is too difficult for patients with PFP, the treatment course can be determined by imaging only [9].

However, the understanding of patellar tracking remains limited. Few studies have described static and dynamic patellofemoral alignments [10–13]. Most studies only described restrictive conditions during non-weight-bearing (NWB) activities in the supine position [14–18]. Especially, one study stated that patellar tracking by unnatural and forced contraction of the quadriceps muscle is performed to assess the impact of joint loading [19, 20]. Another study provided the patellar motion data only during the NWB condition [21]. Research using tracking methods also has challenges. Some tracking marker studies used a metal pin (the tracking marker) inserted into the leg. The metal pin is not part of the patients’ treatment and may be unsafe [12]. One study introduced an electronic tracking device. However, they could not accurately measure the natural patella motion from the patient due to the insecure sensor attachment when moving. A signal sent by a device should be frequently corrected to get reliable data because of the sensor response and signal noise [22]. The devices can provide incorrect measurements during physiologic movement [23].

We previously introduced a biplane fluoroscopy imaging system [19, 20, 24–26]. The system can measure two dual-orthogonal images. The patellar tracking can be visualized on the 2D images and the 3D model. The 3D model is calculated from a 3D scan using MRI. The 3D scan typically requires a lot of measuring time, and the fluoroscopy imaging system is not suitable for application in clinical practice.

The notable features of the limitations in the above studies include: restrictive (posture) conditions, unreal and forceful loading tasks, patient safety concerns, lack of reliability when measuring, and unable to prepare 3D models. To overcome these limitations, we proposed a study for the measurement of the real patellar motion with the following advantages: can be applied in various (posture) conditions, can be used while standing upright (real and active loading tasks), without markers (no safety concerns), without tracking devices (no lack of reliability when measuring), and without the need for pre-information (no need to prepare a prior 3D model). Especially, with the use of a C-arm CT system, we were able to obtain noninvasive measurements of in vivo patellofemoral movements without the use of tracking devices during full weight-bearing conditions in subjects with PF pain and control groups. To place the findings for the full weight-bearing conditions in context, we also provided the measurements during non-weight-bearing conditions in PFP patients and controls.

In this study, we used the 3D knee morphology obtained with this innovative approach to investigate the differences in kinematics of the patellofemoral joint in patients with PFP compared with healthy control subjects. We hypothesize that patients with PFP will have different JCS coordinates with statistical significance (p < 0.05) *in vivo* under NWB and under physiologically relevant WB conditions compared to healthy control subjects.

## Material and methods

### 2.1 Study cohort

This study was approved by Stanford Institutional Review Board (IRB file #20144). All patient data were acquired and used only after written informed consent was obtained. Under the IRB-approved protocol, the study cohort included two groups: a PFP group consisting of 12 females and 6 males (mean age, 31 ± 9 years; mean weight, 68 ± 9 kg) who were treated for more than 6 months, but achieved no symptom improvement, and a control group consisting of 8 females and 10 males (mean age, 39 ± 15 years; mean weight, 71 ± 13 kg) with no symptoms of PFP. Included subjects in the PFP group suffered persistent anterior knee pain for at least three months up to 11 years and reported reproducible pain during at least two of the following physical activities: squatting, stair ascent/descent, kneeling, prolonged sitting, or isometric quadriceps contraction. The measurement conditions for patellar tracking were NWB0° (supine), WB0° (upright), and WB30° (squat). Prior to testing, all study participants received an explanation of the study aims and agreed to participate.

### 2.2 CT image acquisition

**Fig 1:**
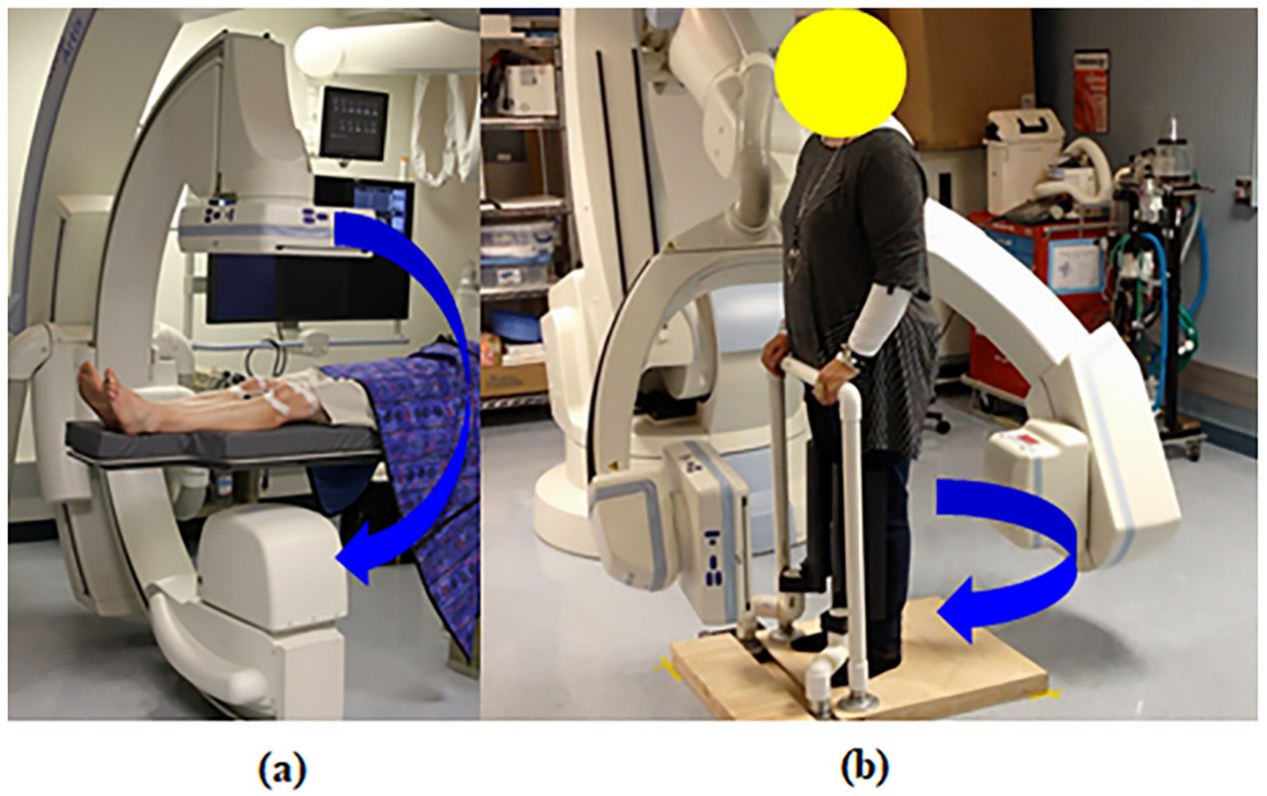
The C-arm CT system (a) in the supine (NWB) and (b) upright (WB) positions under the conditions of 0° and 30° knee flexion. (a) The detector and X-ray source rotated around the knee of the patient in the supine position. (b) The detector and X-ray source rotated around the knee of the patient in the upright posture.

**Fig 2:**
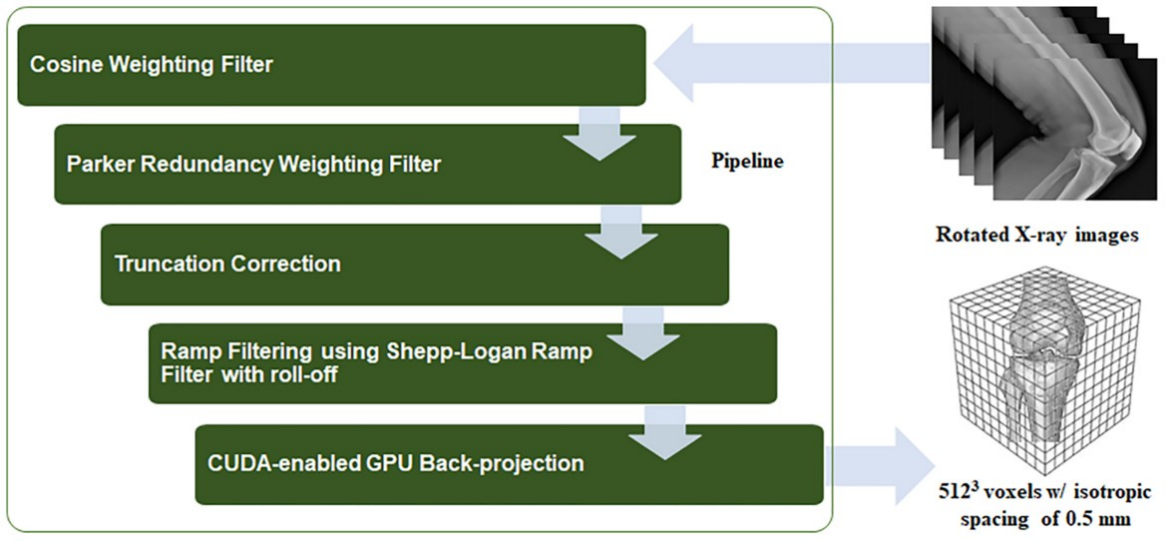
The pipeline of the CONRAD program based on the filtered back projection algorithm. The four steps before the filtered back projection step require accurate CT imaging.

Knee joint alignment under the conditions of WB0° and WB30° was measured on 3D volumetric images acquired with a cone-beam-based C-arm CT imaging system (Artis Zeego; Siemens Healthineers, Forchheim, Germany) as shown in Fig 1. Overexposure correction was applied to obtain saturation-free images by attenuating the X-ray beam on the periphery of an object [27]. The measurement parameters were as follows: photon energy, 80–125 KeV; resolution, 1240 × 960 pixels after 2 × 2 binning; and field-of-view, 300 × 400 mm^2^. The distance between the X-ray source and the patient was 980 mm and that between the patient and the detector was 218 mm. Measurements were acquired with the X-ray source and detector rotating around the patient in circular trajectory (π + fan angle). In total, 248 and 496 images were acquired in the upright (WB) and supine (NWB) positions, respectively. Two-dimensional projection images were reconstructed into a volumetric CT image using a filtered-backprojection method [28–30] implemented in our in-house reconstruction package, named CONRAD (Radiological Sciences Laboratory, Stanford University, Stanford, CA, USA) [31, 32]. A pipeline for the CONRAD software framework for cone-beam imaging is shown in Fig 2.

### 2.3 Patella tracking estimation

**Fig 3:**
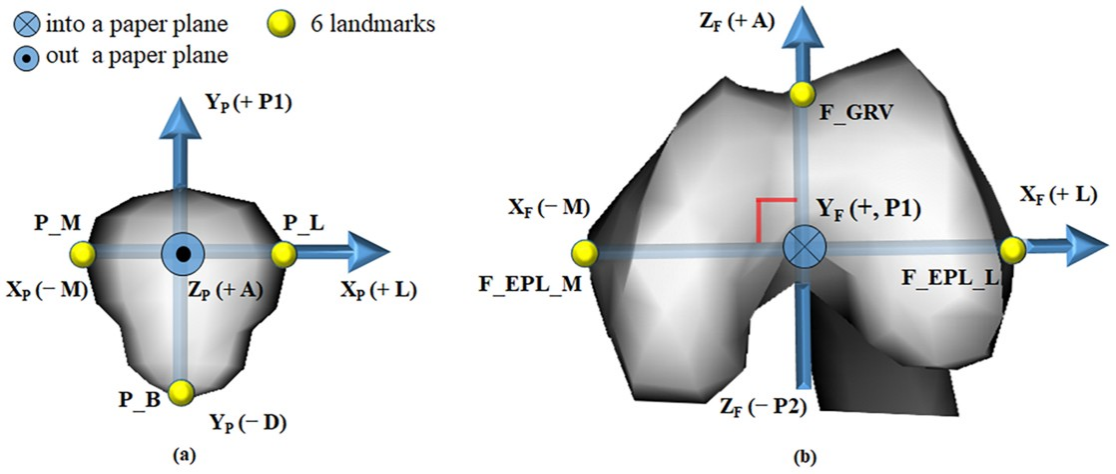
Anatomical landmarks on the patella (a) and femur (b) in left leg. The JCS axes are X_P_ (+ L, − M), X_F_ (+ L, − M), Y_P_ (+ P1, − D), Y_F_ (+ P1, − D), Z_P_ (+ A, − P2), and Z_F_ (+ A, − P2). Landmarks are P_L (patella lateral), P_M (patella medial), P_B (patella bottom), E_EPL_L (femur lateral), E_EPL_M (femur medial), and F_GRV (femur groove). Abbreviations: A, anterior; D, distal; L, lateral; M, medial; P1, proximal; P2, posterior.

**Fig 4:**
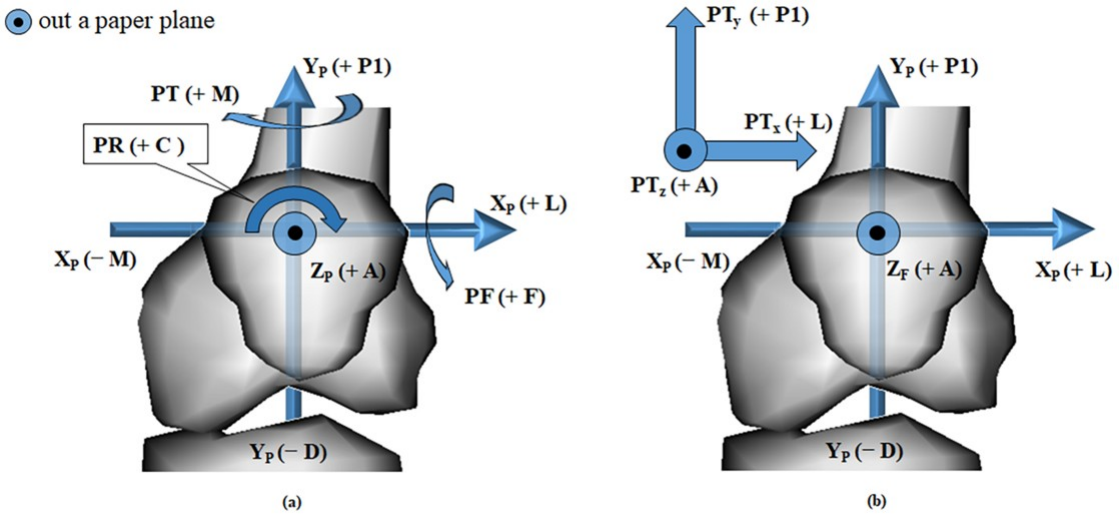
The JCS coordinates of the (a) rotational and (b) translational DoF values of the patella in left leg. The kinematic DoF values on JCS are patellar tilt (PT; + M, − L), patellar flexion (PF; + F, − E), patellar rotation (PR; + C, − CC), patellar lateral-medial shift (PT_x_; + L, − M), patellar proximal-distal shift (PT_y_; + P1, − D), and patellar anterior-posterior shift (PT_z_; + A, − P2). Abbreviations: A, anterior; D, distal; L, lateral; M, medial; P1, proximal; P2, posterior; C, clockwise; CC, counterclockwise; E, extension; F, flexion.

**Fig 5:**
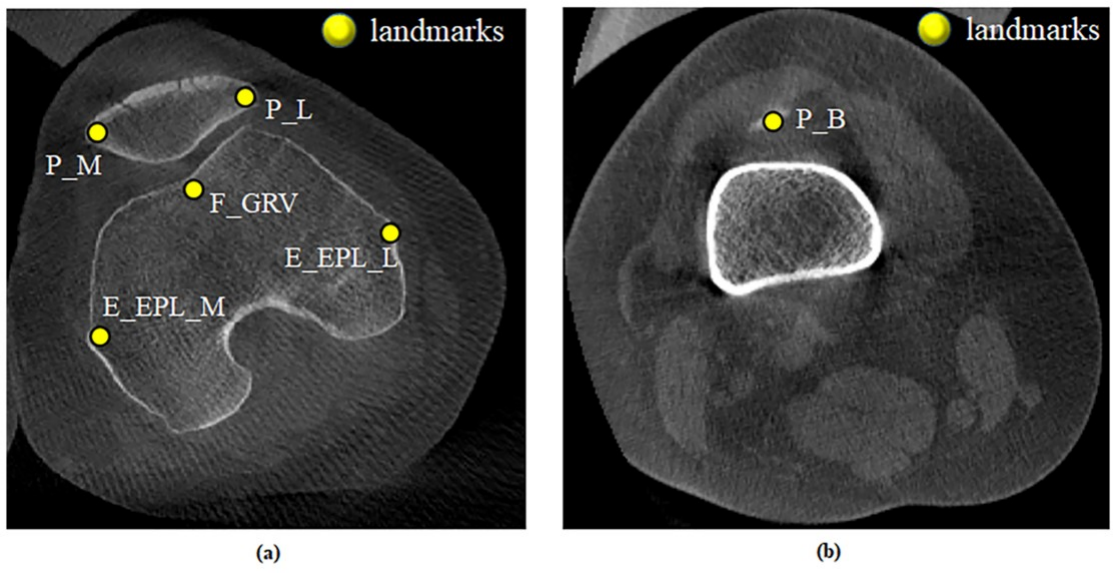
Representative tracking points on CT images. (a) Landmarks on the patella and femur. (b) Landmark on the patella. Abbreviations: P_L, patella lateral; P_M, patella medial; P_B, patella bottom; E_EPL_L, femur lateral; E_EPL_M, femur medial; F_GRV, femur groove.

**Fig 6:**
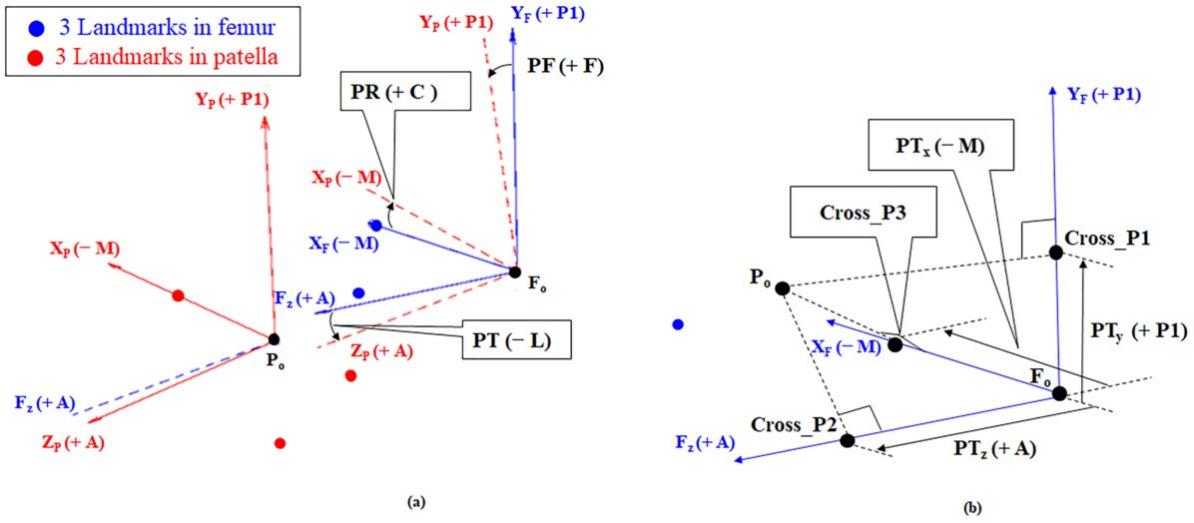
The JCS of the patella and femur and the kinematic DoF values of the six landmarks on the left leg, and the (a) rotational and (b) translational DoF values of the left leg. The kinematic DoF values in the JCS are patellar tilt (PT; + M, − L), patellar flexion (PF; + F, − E), patellar rotation (PR; + C, − CC), patellar lateral-medial shift (PT_x_; + L, − M), patellar proximal-distal shift (PT_y_; + P1, − D), and patellar anterior-posterior shift (PT_z_; + A, − P2). Abbreviations: A, anterior; C, clockwise; CC, counterclockwise; D, distal; E, extension; F, flexion; L, lateral; M, medial; P1, proximal; P2, posterior; PFP, patellofemoral pain; DoF, degrees of freedom.

We set a reference of the joint coordinate system (JCS) as the knee joint of the left leg. Three anatomical landmarks along the *x*, *y*, and *z* axes of the patella and three of the femur were used to establish a JCS. As shown in Fig 3, points P_M (patella medial) and P_L (patella lateral) were the most medial and lateral points with the highest (+) and lowest (−) values on the X_p_ axis. Point P_B (patella bottom) was the lowest point on the patella. Points F_EPL_L (femur lateral) and F_EPL_M (femur medial) were the most lateral and medial points with the highest (+) and lowest (−) values on the X_F_ axis, and point F_GRV (femur groove) had the highest value on the Z_p_ (+) axis. Based on these anatomical landmarks, coordinate axes were established on the patella [X_P_ (+) axis, lateral; X_P_ (−) axis, medial; Y_P_ (+) axis, proximal; Y_P_ (−) axis, distal; Z_P_ (+) axis, anterior; and Z_P_ (−) axis, posterior] and the femur [X_F_ (+) axis, lateral; X_F_ (−) axis, medial; Y_F_ (+) axis, proximal; Y_F_ (−) axis, distal; Z_F_ (+) axis, anterior; and Z_F_ (−) axis, posterior]. Six kinematic degrees of freedom (DoF) values, representing translational and rotational movements of the patella relative to the femur, were derived. According to Fig 4, patellar tilt (PT, °) between the Z_P_ and Z_F_ axis was (+ L) lateral and (− M) medial, patellar flexion (PF, °) between the Y_F_ and Y_P_ axis was (+ F) flexion and (− E) extension, and patellar rotation (PR, °) between the X_P_ and X_F_ axis was (+ C) clockwise and (− CC) counterclockwise. Patellar anterior-posterior shift (PT_z_, mm), a shift of the patellar coordinate system origin projected on the Z_F_ axis, was (+ A) anterior and (− P2) posterior. Patellar proximal-distal shift (PT_y_, mm), the shift projected on the Y_F_ axis, was (+ P1) proximal and (− D) distal. Patellar lateral-medial shift (PT_x_, mm), the shift projected on the X_F_ axis, was (+ L) lateral and (− M) medial. The tracking points on the CT images are shown in Fig 5. Here, we describe the acquirement process for the 3 rotational DOF values for the JCS. For example, let’s consider how to get the PF. The angle size between the Y_F_ and Y_P_ axes is the PF magnitude. Next, we determine the sign of the PF. The PF has a direction of rotation from the Y_F_ axis (femur) to the Y_P_ axis (patella). The rotation direction (Y_F_ to Y_P_) corresponds to one of two rotation directions (+ flexion, − extension) in Fig 6a. The sign in the corresponding direction becomes the sign of the PF value. The sign of the PF is + (flexion). We can determine the magnitude and sign of the PF. We can find the rest of the variables in the same way.

Next, we will describe the acquirement process of the 3 shifted DOF values for the JCS. For example, let’s consider how to get Z_F_. We find a projection point (Cross_P2) of P_o_ in the extension line of the Z_F_ axis. The projection is the orthogonal condition. The distance between the projection point and F_o_ is the PT_z_ magnitude. We determine the sign of the PT_z_. The direction from F_o_ to Cross_P2 (F_o_ to Cross_P2) corresponds to one of two directions (+ anterior,—posterior) in Fig 6b. The sign of the PT_z_ is + (anterior). We can determine the magnitude and sign of the PT_z_. We can find the rest of the variables in the same way. The DoF values on JCS were computed by the codes implemented in MATLAB R2015b (MathWorks, Natick, MA, USA), as shown in Fig 6. Additionally, to identify the statistical differences between the subjects and the PFP patients, we have summarized the p-values from t-tests in Table 2 for the comparisons between the patients and the controls.

### 2.4 Statistical analysis

We evaluated the kinematic difference between the two groups (patients with patellofemoral pain and controls) using five statistical tests: 1. Unpaired t-test (Table 2), 2. One way analysis of variance (ANOVA) (S1 Table), 3. Wilcoxon rank sum (S1 Table), 4. Mann-Whitney test (S1 Table), and 5. Kolmogorov-Smirnov test (S1 Table). A p-value less than 0.05 was considered statistically significant and could differentiate the kinematics between the two groups. ANOVA was implemented using the Python program language; all other statistical methods were implemented using MATLAB.

## Results

### 3.1 Patella tracking parameter analysis

**Fig 7:**
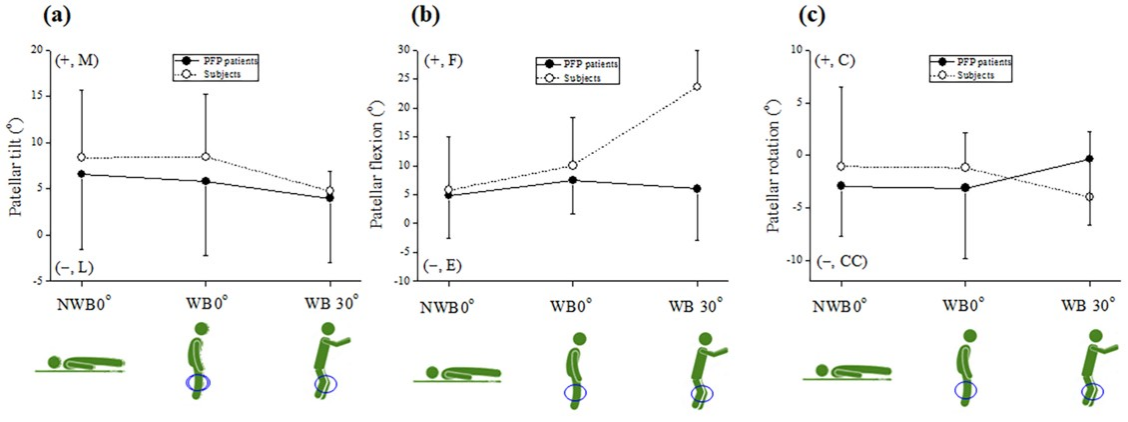
Patellar tilt (a), flexion (b), rotation (c), and mean DoF (± SD) values under three conditions: NWB0° (supine), WB0° (upright), and WB30° (squat). Abbreviations: C, clockwise; CC, counterclockwise; E, extension; F, flexion; L, lateral; M, medial; PFP, patellofemoral pain; DoF, degrees of freedom; SD, Standard deviation.

**Fig 8:**
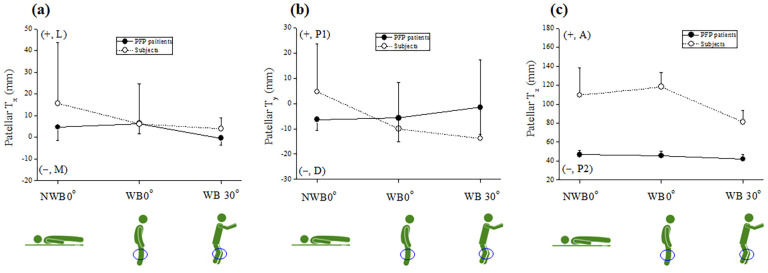
Patellar medial-lateral shift (PT_x_) (a), proximal-distal shift (PT_y_) (b), anterior-posterior shift (PT_z_) (c), and mean DoF (± SD) values under three conditions: NWB0° (supine), WB0° (upright), and WB30° (squat). Abbreviations: A, anterior; D, distal; L, lateral; M, medial; P1, proximal; P2, posterior; PFP, patellofemoral pain; DoF, degrees of freedom; SD, Standard deviation.

The mean kinematic DoF (± standard deviation, SD) values include patellar medial-lateral shift (PT_x_, mm), proximal-distal shift (PT_y_, mm), anterior-posterior shift (PT_z_, mm), tilt (PT, °), flexion (PF, °), and rotation (PR, °), respectively. The three conditions were NWB0°, WB0°, and WB30°, respectively. The obtained DoF values are shown in Table 1 and Figs 7 and 8.

**Table 1:** The mean DoF (± SD) values of three conditions (NWB0°, WB0°, and WB30°) in both groups (control subjects and patients with PFP).

**JCS coordinates**	**Control subjects**		
	**NWB0°**	**WB0°**	**WB30°**
Patellar tilt (°)	8.36 (±7.3)	8.44 (±6.76)	4.75 (±2.1)
Patellar flexion (°)	5.75 (±9.3)	10.04 (±8.38)	23.63 (±6.35)
Patellar rotation (°)	-1.07 (±7.59)	-1.21 (±3.37)	-3.98 (±6.26)
Patellar lateral-medial shift (mm)	15.65 (±28.15)	6.21(±18.52)	3.96 (±5)
Patellar proximal-distal shift (mm)	4.6 (±19.13)	-10.07 (±18.33)	-13.84 (±31.21)
Patellar anterior-posterior shift (mm)	109.47 (±28.86)	118.05 (±15.56)	81.09 (±12.46)
	**Patellofemoral pain patients**		
	**NWB0°**	**WB0°**	**WB30°**
Patellar tilt (°)	6.58 (±8.11)	5.82 (±8.05)	3.96 (±6.93)
Patellar flexion (°)	4.82 (±7.4)	7.48 (±5.76)	6.01 (±8.95)
Patellar rotation (°)	-2.94 (±4.75)	-3.12 (±6.72)	-0.38 (±6.24)
Patellar lateral-medial shift (mm)	4.73 (±6.22)	6.39 (±4.64)	-0.44 (±3.15)
Patellar proximal-distal shift (mm)	-6.36 (±4.36)	-5.78 (±9.28)	-1.47 (±10.83)
Patellar anterior-posterior shift (mm)	46.58 (±4.91)	45.55 (±4.82)	41.92 (±4.65)

^a^NWB0° (supine), NWB at 0° knee flexion; ^b^WB0°(upright), WB at 0° knee flexion; ^c^WB30°(squat), WB at 0° 30° knee flexion; ^d^JCS, joint coordinate system (n = 18/group), Abbreviations: PFP, patellofemoral pain; DoF, degrees of freedom; SD, Standard deviation.

As shown in Table 2, the statistical analysis based on the unpaired t-test resulted in five different JCS coordinates with significant differences (p < 0.05) between patients with PFP and control subjects. The statistically significant JCS coordinates included patellar anterior-posterior shift under all three loading conditions, patellar proximal-distal shift under NWB0°, and patellar flexion under WB30°. These statistically significant results generally corresponded well to the results based on the other four representative statistical methods, except for the Kolmogorov-Smirnov test, as shown in S1 Table. The Kolmogorov-Smirnov test identified two additional JCS coordinates with significant differences, namely the patellar rotation under NWB0° and the patellar proximal-distal shift under WB30°.

**Table 2:** The p-values (Unpaired t-test) of three conditions (NWB0°, WB0°, and WB30°) in control subjects and patients with PFP. The p-values highlighted in boldface indicate statistical significance (p<0.05).

	P-values (Patients with PFP *vs* Controls)		
JCS coordinates	Unpaired t-test		
	NWB0°	WB0°	WB30°
Patellar tilt (°)	0.527	0.358	0.716
Patellar flexion (°)	0.759	0.365	***0*.*003***
Patellar rotation (°)	0.422	0.343	0.357
Patellar lateral-medial shift (mm)	0.162	0.974	0.176
Patellar proximal-distal shift (mm)	***0*.*046***	0.456	0.489
Patellar anterior-posterior shift (mm)	***<0*.*001***	***<0*.*001***	***0*.*007***

^a^NWB0° (supine), NWB at 0° knee flexion; ^b^WB0°(upright), WB at 0° knee flexion; ^c^WB30°(squat), WB at 0° 30° knee flexion; ^d^JCS, joint coordinate system (n = 18/group). Abbreviations: PFP, patellofemoral pain.

Fig 7a–7c show the rotational DoF values for patellar tilt (PT, °), flexion (PF, °), and rotation (PR, °). As shown in Fig 7a, the PT values of the control group were similar during NWB0° (+8.36° ± 7.3°) and WB0° (+8.44° ± 6.76°) toward the medial direction (+ M) and were tilted during WB0° (+8.44° ± 6.76°) to WB30° (+4.75° ± 2.1°) toward the lateral direction (− L). The PT values of the PFP group differed during NWB0° (+6.58° ± 8.11°), WB0° (+5.82° ± 8.05°), and WB30° (+3.96° ± 6.93°).

As shown in Fig 7b, the PF values of the subjects differed during NWB0° (+5.75° ± 9.3°) and WB0° (+10.04° ± 8.38°). The PF values of the control group were dramatically tilted during WB0° (+10.04° ± 8.38°) to WB30° (+26.63° ± 6.35°) toward the flexion direction (+ F). On the other hand, the PT values of the patients differed during NWB0° (+4.82° ± 7.4°), WB0° (+7.48° ± 5.76°), and WB30° (+6.01° ± 8.95°). The PF values of the PFP group changed little, as compared with the control group.

As shown in Fig 7c, the PR values of the control group were similar during NWB0° (−1.07° ± 7.59°) and WB0° (−1.21° ± 3.37°), and were also similar in the PFP group during NWB0° (−2.94° ± 4.75°) and WB0° (−3.12° ± 6.72°). However, each PR value of the PFP and control groups were reversed during WB0° to WB30°.

Fig 8a–8c show the DoF values of patellar medial-lateral (PT_x_, mm), proximal-distal (PT_y_, mm), and anterior-posterior shift (PT_z_, mm). In Fig 8a, the PT_x_ values of the subjects differed during NWB0° (+15.65 ± 28.15 mm) and WB0° (+6.21 ± 18.52 mm), and were tilted from WB0° (+6.21 ± 18.52 mm) and WB30° (+3.96 ± 5 mm) toward the medial direction (− M). The PT_x_ values of the PFP group differed during NWB0° (4.73 ± 6.22 mm) and WB0° (6.39 ± 4.64 mm), and were tilted from WB0° (6.39 ± 4.64 mm) to WB30° (−0.44 ± 3.15 mm) toward the medial direction (− M). In Fig 8b, The PT_y_ values of the subjects differed during NWB0° (+4.6 ± 19.13 mm) and WB0° (−10.07 ± 18.33 mm), and were tilted from WB0° (−10.07 ± 18.33 mm) to WB30° (−13.84 ± 31.21 mm) toward the distal direction (− D). The PT_y_ values of the PFP group differed during NWB0° (−6.36 ± 4.36 mm) and WB0° (−5.78 ± 9.28 mm), and were tilted from WB0° (−5.78 ± 9.28 mm) to WB30° (−1.47 ± 10.83 mm) toward the proximal direction (+ P1). In Fig 8c, the PT_z_ values of the subjects differed during NWB0° (109.47 ± 28.86 mm) and WB0° (118.05 ± 15.56 mm), and were tilted from WB0° (118.05 ± 15.56 mm) to WB30° (81.09 ± 12.46 mm) toward the posterior direction (− P2). The PT_z_ values of the PFP group differed during NWB0° (46.58 ± 4.91 mm) and WB0° (45.55 ± 4.82 mm), and were somewhat tilted from WB0° (45.55 ± 4.82 mm) to WB30° (41.92 ± 4.65 mm) toward the posterior direction (− P2). Overall, there were significant differences in the PT_z_ values between the PFP and control groups.

**Fig 9:**
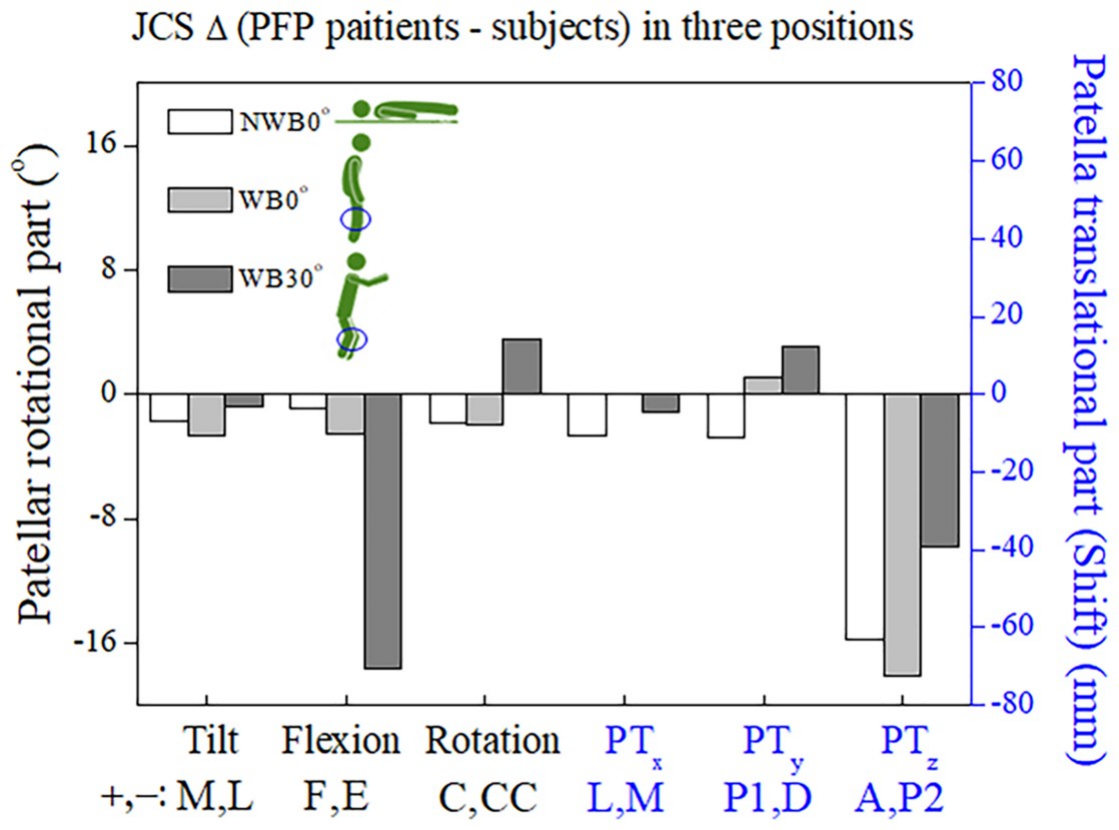
Differences in mean DoF values between the PFP and control groups under three conditions: NWB0° (supine), WB0° (upright), and WB30° (squat). Patellar medial-lateral shift (PT_x_, mm), proximal-distal shift (PT_y_, mm), anterior-posterior shift (PT_z_, mm), tilt (PT, °), flexion (PF, °), and rotation (PR, °). Abbreviations: A, anterior; C, clockwise; CC, counterclockwise; D, distal; E, extension; F, flexion; L, lateral; M, medial; P1, proximal; P2, posterior; DoF, degrees of freedom; PFP, patellofemoral pain.

Fig 9 shows the differences in the DoF values between the PFP patients and healthy subjects. The DoF values showed the largest translational differences in the PF during WB30° with extension (− E: − 17.62°) (p < 0.01) and the largest rotational differences in the PT_z_ during WB0° toward the posterior direction (− P2: − 72.5‬0 mm) (p < 0.01).

The PFP trends between males and females are shown in S1 and S2 Figs. The largest differences between females and males in the control group were observed in the PF (6.29°) and PT_y_ (36.42 mm) values during WB30°. The greatest differences between males and females in the PFP group were observed in PF values (−17.25°) during WB30° and PT_z_ values (−71.47 mm) during WB0°.

## Discussion

In this study, we hypothesized that patients with PFP will show different JCS coordinates in vivo under NWB and under physiologically relevant WB conditions compared to healthy control subjects. This hypothesis was supported by the findings presented in the current study. The data for the 18 patients confirmed that the PF during WB30°, the PT_y_ during NWB0°, and the PT_z_ during NWB0°, WB0°, and WB30° were statistically different (p<0.05) between the patients with PFP and healthy controls. Of the five statistically significant JCS coordinates presented, two p-values (PF and PT_z_ during WB30°) were less than 0.01 and the other two (PT_z_ during NWB0°, WB0°) were less than 0.001 (Table 2). Given that p<0.001 is generally considered to indicate high statistical significance, the significant JCS coordinates presented in this study can serve as accurate biomarkers to diagnose knee conditions.

A study by Bruno et al. [33] reported that despite contraction of the quadriceps, there were obvious differences in the lateral translation of the patella relative to the femur during WB0° in the upright position and NWB0° in the supine position. Hence, we focused on patellar tracking during the following conditions: NWB0°, WB0°, and WB30°.

Some reports have provided measurements during unrealistic conditions, such as PFP evaluation in the supine position only [34] or while leaning on the equipment during the loading task [23]. For example, in a study by Esfandiarpour et al. [19], the lunge test was performed with one leg supported on the ground, while the knee of the other leg was flexed at 90°. The lunge, supine, and leaning tests differ from actual conditions that involve squatting or straightening to mimic movements performed in daily activities. Hence, the conditions used in several previous studies were controversial and not practical for the study of PFP. Therefore, clinically relevant weight-bearing conditions were employed in the present study (WB0° and WB30°) for the diagnosis of PFP.

Esfandiarpour et al. [19] reported that the PT values of the PFP patients during NWB0° were laterally tilted (− L) as compared with those of the control group due to the stabilization by the retinacula and ligaments, as well as the articular geometry. In the present study, the PT values during NWB0° of the PFP and control groups (p<0.53) were +8.36° ± 7.3° and +6.58° ± 8.11°, respectively. On the other hand, the PT values of the PFP group during WB0° (p<0.36) and WB30° (p<0.72) showed abnormal lateral patellar tilt as compared with the control group. The PT values of the control group remained relatively constant. Regarding the difference between the PT values of the PFP and control groups, our results are generally consistent with the trends in value change compared to the referenced study [19]. However, our values are different from the referenced study’s values.

Draper et al. [23] reported that the PT_x_ values of the PFP group were lower during WB0° than NWB0°. In the cited study, patellar motion is generally generated by quadriceps contraction. The quadriceps contraction has a gap between the WB0° (weight-bearing) and NWB0° (non-weight-bearing). The quadriceps contraction during NWB0° shows unbalanced activation by the vastus medialis obliquus. However, the quadriceps contraction during WB0° doesn’t have unbalanced activation in the quadriceps. The quadriceps contraction during NWB0°, due to unbalanced quadriceps motion, causes more lateral patella translation compare to that during WB0°.

Conversely, we have a different opinion than the authors of the cited study. Abnormal pressure on the patella surface by wrong alignment of the patella causes pain when the patella isn’t precisely in the center of the groove of the femur. To avoid the pain, patients generally try to move the patella to a region with less pain. A laterally translated patella typically is not located in the center. Therefore, the patient unconsciously contracts muscles that makes it move in the opposite direction (medial translation) to alleviate the pain. The patella can move relative to the medial direction. The PT_x_ values of the PFP group were higher during WB0° than NWB0°. Unlike the cited study, our PT_x_ values of the PFP group were greater during WB0° than NWB0° (6.39 ± 4.64 vs. 4.73 ± 6.22 mm, respectively).

Additionally, our NWB0° are different from those in the cited study. In our NWB0°, the whole body is in contact with the ground. However, in the cited study’s NWB0°, the upper part of the body is in contact with the ground, but the legs are not. The patella indirectly receives the load from the lower leg. The cited study’s condition is not actually NWB0°. Our NWB0° is a more suitable NWB condition than the cited study’s NWB0°.

Esfandiarpour et al. [19] also reported a difference in PR values between the PFP and control groups during NWB0° and WB0° of about 40°, while in the present study, there was a difference in the cross-flexion angle of about 10° between NWB0° and WB0°.

We focused on the resulting patellar flexion (PF, °), patellar anterior-posterior shift (PT_z_, mm), and patellar proximal-distal shift (PT_y_, mm) values. First, as shown in Figs 8b, 9b and 9c, the PF, PT_z_, and PT_y_ values during WB0° to WB30° were significantly changed in the control group. In contrast, those of the PFP group during WB0° to WB30° changed very little. Second, as shown in Fig 7b, there were differences in PF value gaps during NWB0° vs. WB0° with the greatest difference during WB30°. Third, as shown in Fig 8b, the PT_y_ values during NWB0° and WB 0° were reversed the PFP and control group. Fourth, as shown in Fig 8c, there were differences in PT_z_ values during NWB0°, WB0°, and WB30° between the PFP and control groups. Lastly, as shown in Fig 9, the largest differences were observed in the values of PF during WB30° (− E: − 17.62°) (*p* < 0.01) and PT_z_ during WB0° (− P2: − 72.5‬0 mm) (*p* < 0.01) between the PFP patients and healthy subjects which suggests that PF during WB30° and PT_z_ during WB0° can be used as diagnostic biomarkers for identifying patients with PFP.

Carlson et al. showed that a strongly contracted quadriceps reduce the bony constraint on the patella, causing the patella to deviate from normal tracking along the femoral groove [34]. Especially, in weight-bearing conditions, an activation imbalance of quadriceps causes the abnormal action of the patella in the PFP group [19]. Previous studies have speculated that the cause of the activation imbalance of quadriceps might be that the muscles in the PFP group adapted to reduce the concentrated joint stress and consequently reduce pain, increasing the area of patellofemoral contact [35, 36]. Moreover, the patellofemoral joint contact area increases with PF [37] and PF is highly corrected with PT_z_. Our results showed that the largest differences in DoF values between the PFP and control group was observed in PF during WB30° (− E: − 17.62°) (*p* < 0.01) and PT_z_ during WB0° (− P2: − 72.5‬0 mm) (*p* < 0.01), which suggests that the PFP group presumably adapted to avoid knee pain. To further understand the origin of knee pain, we suggest evaluation of the patellofemoral contact area between the patella and the femur on CT images.

There were several limitations to this study: (1) factors involved with patellar movement, such as joint morphology, knee muscle activity, and tendon function [23] were not considered; (2) PFP should be accurately distinguished from other types of anterior knee pain, such as patellar tendinopathy [35]; (3) other clinical causes of knee pain attributed to rheumatologic or neurologic pathologies should be ruled out [35]; (4) although patellar maltracking is sometimes related to the condition of the peripatellar fat pads [38], the effects of the peripatellar fat pads were not considered; and (5) we did not consider the function of the patellofemoral joint with more common causes of PFP such as when walking or using the stairs. However, Bruno et al. [33] reported that it is not always possible to apply more than 25% of the body weight with knee flexion of 90°. (6) It is imperative to evaluate the entire kinetic chain in a comprehensive approach to the treatment of PFP [39]. We should keep in mind the dynamic relationships of the hip and ankle joints with the knee joints. These relationships are complex because the hip and ankle joints can affect the knee joints. For example, excessive rearfoot eversion is a factor correlated with the development and persistence of PFP in some cases. For patients with chronic PFP, psychological factors should be considered [40]. Therefore, future research should consider the combined effects of various factors under appropriate circumstances in PFP patients. We will plan to investigate the patellofemoral contact area between the patella and femur in a future study.

## Conclusion

This study proposed an innovative approach to accurately diagnose patellar motion such as patellar maltracking in PFP patients by using a clinical C-arm CT scanner capable of acquiring a volumetric CT image of patients under realistic weight-bearing (WB0° and WB30°) and supine, non-weight-bearing conditions (NWB0°). When comparing the patients with PFP and control subjects, significant differences (p < 0.05) were observed for patellar proximal-distal shift (PT_y_) during NWB0°, patella flexion (PF) during WB30°, and patella anterior-posterior shift (PT_z_) during NWB0°, WB0°, and WB30° on the CT scan. In particular, the rotational and translational differences (JCS Δ = patients with PFP—controls) in DoF values were clearly seen in the PF during WB30° (−17.62 °, extension) (p < 0.001) and the PT_z_ during WB0° (−72.5‬0 mm, posterior) (p = 0.007). We showed that the action was revealed by the PT_y_ during NWB0°, the PF during WB30°, and the PT_z_ during NWB0°, WB0°, and WB30°, which could be used as diagnostic biomarkers for identifying patients with PFP. Our results provide new insights toward an improved understanding of patellofemoral joint movement during non- and weight-bearing conditions. The proposed method is an effective adjunct for clinical diagnosis before surgery and to help plan rehabilitation strategies.

## Supporting information

S1 Fig Mean DoF values (± SD) of females vs. males in the PFP group under three conditions: NWB0° (supine), WB0° (upright), and WB30° (squat). (a) Patellar tilt (PT, °), flexion (PF, °), and rotation (PR, °). (b) Patellar medial-lateral shift (PT_x_, mm), proximal-distal shift (PT_y_, mm), and anterior-posterior shift (PT_z_, mm). Abbreviations: A, anterior; C, clockwise; CC, counterclockwise; D, distal; E, extension; F, flexion; L, lateral; M, medial; P1, proximal; P2, posterior; PFP, patellofemoral pain; DoF, degrees of freedom; SD, Standard deviation.
(TIF)

S2 Fig Mean DoF values (± SD) of females vs. males in the control group under three conditions: NWB0° (supine), WB0° (upright), and WB30° (squat). (a) Patellar tilt (PT, °), flexion (PF, °), and rotation (PR, °). (b) Patellar medial-lateral shift (PT_x_, mm), proximal-distal shift (PT_y_, mm), and anterior-posterior shift (PT_z_, mm). Abbreviations: A, anterior; C, clockwise; CC, counterclockwise; D, distal; E, extension; F, flexion; L, lateral; M, medial; P1, proximal; P2, posterior; DoF, degrees of freedom; SD, Standard deviation.
(TIF)

S1 Table The p-values (one-way ANOVA, Wilcoxon rank sum test, Mann-Whitney U-test, and Kolmogorov-Smirnov test) of three conditions (NWB0°, WB0°, and WB30°) in subjects and patients with PFP. The p-values highlighted in boldface indicate statistical significance (p<0.05).
(DOCX)
