# Supplementary figures and images for: The effect of patellofemoral pain syndrome on patellofemoral joint kinematics under upright weight-bearing conditions
Source: PLoS One. 2020 Sep 30;15(9):e0239907. doi: 10.1371/journal.pone.0239907 (PMC7526904; doi:10.1371/journal.pone.0239907)

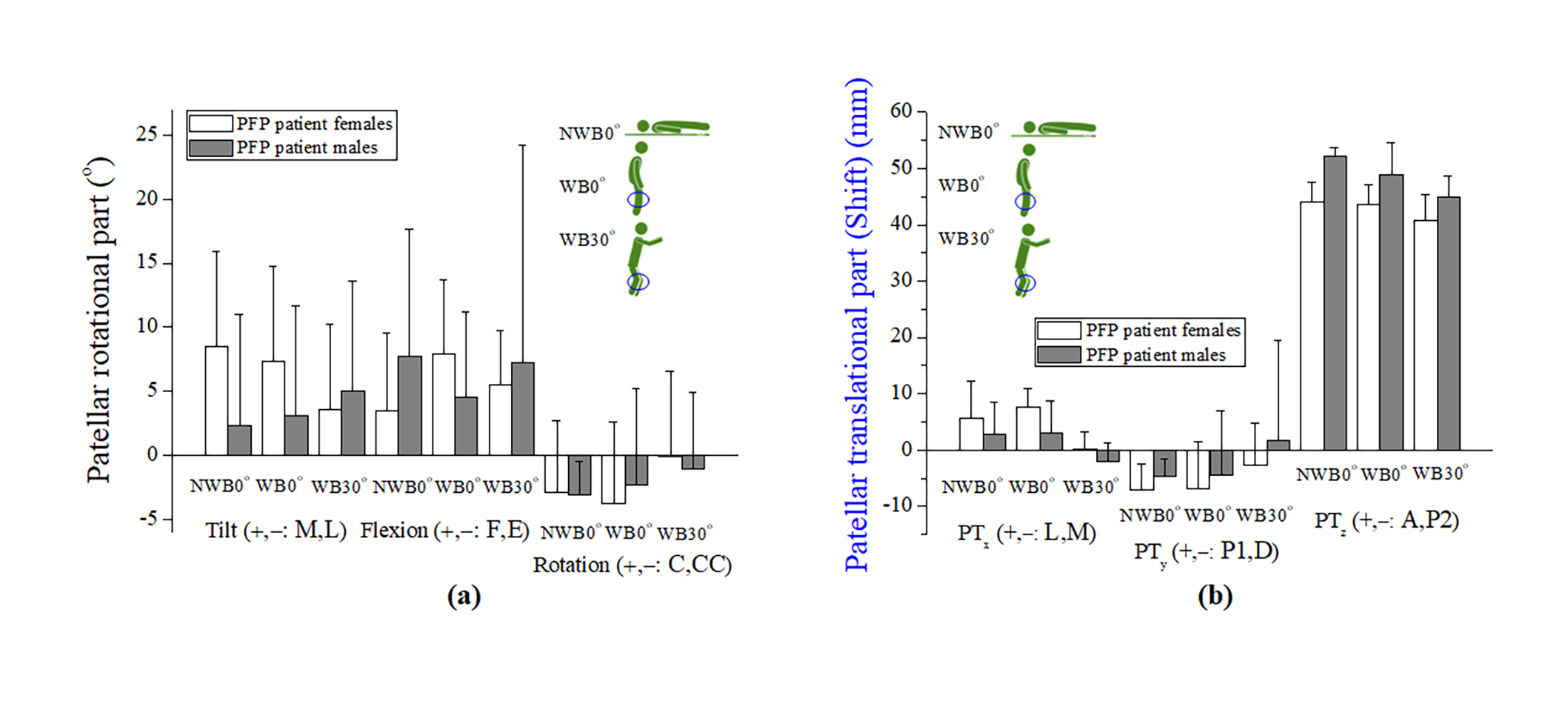

Supplement: S1 Fig — (a) Patellar tilt (PT, °), flexion (PF, °), and rotation (PR, °). (b) Patellar medial-lateral shift (PTx, mm), proximal-distal shift (PTy, mm), and anterior-posterior shift (PTz, mm). Abbreviations: A, anterior; C, clockwise; CC, counterclockwise; D, distal; E, extension; F, flexion; L, lateral; M, medial; P1, proximal; P2, posterior; PFP, patellofemoral pain; DoF, degrees of freedom; SD, Standard deviation. (TIF) [file pone.0239907.s001.tif]

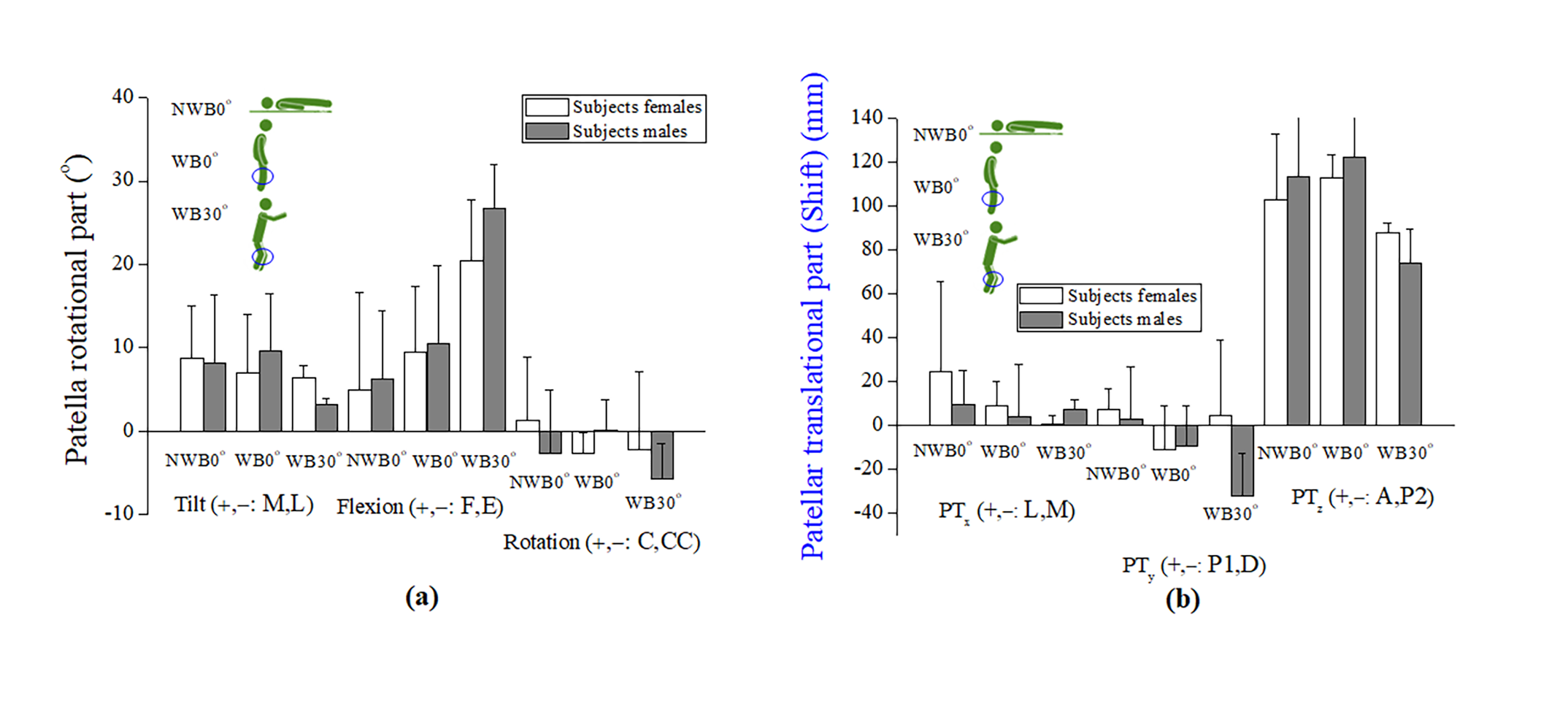

Supplement: S2 Fig — (a) Patellar tilt (PT, °), flexion (PF, °), and rotation (PR, °). (b) Patellar medial-lateral shift (PTx, mm), proximal-distal shift (PTy, mm), and anterior-posterior shift (PTz, mm). Abbreviations: A, anterior; C, clockwise; CC, counterclockwise; D, distal; E, extension; F, flexion; L, lateral; M, medial; P1, proximal; P2, posterior; DoF, degrees of freedom; SD, Standard deviation. (TIF) [file pone.0239907.s002.tif]
