# Supplementary material for: The effect of patellofemoral pain syndrome on patellofemoral joint kinematics under upright weight-bearing conditions
Source: PLoS One. 2020 Sep 30;15(9):e0239907. doi: 10.1371/journal.pone.0239907 (PMC7526904; doi:10.1371/journal.pone.0239907)
Supplement: S1 Table — The p-values highlighted in boldface indicate statistical significance (p<0.05). (DOCX) [file pone.0239907.s003.docx]

**S1 Table. The p-values (one-way ANOVA, Wilcoxon rank sum test, Mann-Whitney U-test, and Kolmogorov-Smirnov test) of three conditions (NWB0°, WB0°, and WB30°) in subjects and patients with PFP. The p-values highlighted in boldface indicate statistical significance (p<0.05).**

|  | **P-values (Patients with PFP *vs* Controls)** | | |
| --- | --- | --- | --- |
| **JCS coordinates** | **One way ANOVA** | | |
|  | **NWB0°** | **WB0°** | **WB30°** |
| Patellar tilt (°) | 0.528 | 0.364 | 0.828 |
| Patellar flexion (°) | 0.758 | 0.350 | ***0.002*** |
| Patellar rotation (°) | 0.414 | 0.362 | 0.323 |
| Patellar lateral-medial shift (mm) | 0.141 | 0.972 | 0.045 |
| Patellar proximal-distal shift (mm) | ***0.033*** | 0.432 | 0.209 |
| Patellar anterior-posterior shift (mm) | ***<0.001*** | ***<0.001*** | ***<0.001*** |
|  | **Wilcoxon rank sum test** | | |
|  | **NWB0°** | **WB0°** | **WB30°** |
| Patellar tilt (°) | 0.565 | 0.406 | 0.939 |
| Patellar flexion (°) | 0.619 | 0.406 | ***0.008*** |
| Patellar rotation (°) | 0.137 | 0.675 | 0.369 |
| Patellar lateral-medial shift (mm) | 0.333 | 0.919 | 0.119 |
| Patellar proximal-distal shift (mm) | ***0.007*** | 0.991 | 0.149 |
| Patellar anterior-posterior shift (mm) | ***<0.001*** | ***<0.001*** | ***<0.001*** |
|  | **Mann-Whitney U-test** | | |
|  | **NWB0°** | **WB0°** | **WB30°** |
| Patellar tilt (°) | 0.566 | 0.407 | 0.939 |
| Patellar flexion (°) | 0.621 | 0.407 | ***0.008*** |
| Patellar rotation (°) | 0.138 | 0.678 | 0.369 |
| Patellar lateral-medial shift (mm) | 0.333 | 0.927 | 0.119 |
| Patellar proximal-distal shift (mm) | ***0.009*** | 1.000 | 0.149 |
| Patellar anterior-posterior shift (mm) | ***<0.001*** | ***<0.001*** | ***<0.001*** |
|  | **Kolmogorov Smirnov** | | |
|  | **NWB0°** | **WB0°** | **WB30°** |
| Patellar tilt (°) | 0.264 | 0.514 | 0.490 |
| Patellar flexion (°) | 0.773 | 0.367 | ***0.003*** |
| Patellar rotation (°) | ***0.035*** | 0.632 | 0.227 |
| Patellar lateral-medial shift (mm) | 0.278 | 0.173 | 0.123 |
| Patellar proximal-distal shift (mm) | ***0.004*** | 0.406 | ***0.028*** |
| Patellar anterior-posterior shift (mm) | ***<0.001*** | ***<0.001*** | ***<0.001*** |

^a^NWB0° (supine), NWB at 0° knee flexion; ^b^WB0°(upright), WB at 0° knee flexion; ^c^WB30°(squat), WB at 0° 30° knee flexion; ^d^JCS, joint coordinate system (n = 18/group). Abbreviations: PFP, patellofemoral pain.
